# Nonlinear Association of Daily Sitting Time With Depression Risk in US Adults: A Cross‐Sectional Study

**DOI:** 10.1155/bn/7167267

**Published:** 2026-03-04

**Authors:** Zhimao Cai, Hualang Cai, Rui Jia, Hui Peng, Youlian Luo, Jiashuang Lin, Ye Ye, Sixia Chen, Yueqian Wang, Rourou Li, Mengjia Chen, Weifeng Chen

**Affiliations:** ^1^ Department of General Medicine, Shenzhen Second People′s Hospital, The First Affiliated Hospital of Shenzhen University, Shenzhen, Guangdong, China, szu.edu.cn; ^2^ Shenzhen University Health Science Center, Shenzhen, Guangdong, China, szu.edu.cn; ^3^ Department of Hepatology, National Clinical Research Center for Infectious Diseases, The Third People′s Hospital of Shenzhen, Shenzhen, Guangdong, China, szdsyy.com; ^4^ Yanchuan Community Health Service Center, Songgang People′s Hospital Baoan District, Shenzhen, Guangdong, China

**Keywords:** cross-sectional study, daily sitting time, depression, NHANES, nonlinear association

## Abstract

**Background:**

Previous studies have examined the relationship between daily sitting time and depression, yet the specific dose–response relationship remains unclear.

**Methods:**

This study analyzed data from 29,691 participants in the 2007–2018 National Health and Nutrition Examination Survey (NHANES). To delve into the possible nonlinear link between daily sitting time and depression, smooth curve fitting and threshold effect analysis were utilized in the study. Information on daily sitting time was collected through questionnaires, and the Patient Health Questionnaire‐9 (PHQ‐9) was employed to measure depression.

**Results:**

In the fully adjusted Model 3, each additional hour of sitting was associated with a 5% increase in the risk of depression (95% CI: 1.02, 1.07, *p* = 0.0002). When categorizing daily sitting time, participants who sat for 8 h or more daily exhibited a significantly higher risk of depression across all models. In Model 3, this group had an odds ratio (OR) of 1.37 (95% CI: 1.12, 1.69, *p* = 0.0039) compared with the reference group sitting for less than 4 h. The threshold effect analysis identified 7 h as the inflection point. Below this threshold, no significant correlation was observed. In contrast, when daily sitting time exceeded the threshold, there was a significant increase in the risk of depression, with an OR of 1.07 (95% CI: 1.01, 1.12, *p* = 0.0184).

**Conclusions:**

A nonlinear association exists between daily sitting time and depression risk in US adults. However, additional research is required to further validate this finding.

## 1. Introduction

Depression poses a severe and escalating challenge to global health, afflicting hundreds of millions of people and imposing a substantial economic burden on healthcare systems worldwide [1]. The detrimental effects of depression extend beyond the individual, influencing overall societal productivity and well‐being. As a widely recognized mental health disorder, it profoundly diminishes quality of life and disrupts daily functioning [2, 3]. According to estimates by the World Health Organization (WHO), depressive disorders are predicted to become one of the leading contributors to global disease burden, accounting for approximately 4.3% of the total burden by 2030. Currently, it affects over 300 million individuals globally [4]. Recent investigations have increasingly focused on the modifiable lifestyle factors associated with depression, particularly an emerging body of evidence linking sedentary behavior to increased depression risk [5, 6].

Sedentary behavior is characterized by prolonged periods of inactivity and has been implicated in numerous health issues, including depression. Research indicates that higher amounts of daily sitting time increase the risk of developing depressive symptoms [7, 8]. Prolonged periods of sitting are associated with reduced physical activity (PA) levels, metabolic disruption, and heightened inflammatory responses, all of which may be associated with the emergence or worsening of depressive symptoms [9–12]. Comprehensive studies have reported a statistically significant association between increased daily sitting time and elevated risk of depression, suggesting a direct correlation between sedentary behavior and mental health outcomes [13, 14]. This relationship highlights the necessity of considering lifestyle patterns when addressing mental health interventions, particularly in populations at risk for depression [15]. However, the previous body of research has largely focused on linear relationships between sedentary time and depression; the specific dose–response relationship remains unclear [16].

The National Health and Nutrition Examination Survey (NHANES) offers a valuable platform for examining this relationship using a nationally representative dataset. This study is designed to investigate the association between daily sitting time and depression.

## 2. Methods

### 2.1. Study Design and Population

This research employs a cross‐sectional analytical framework using data from the 2007 to 2018 cycles of the NHANES, collaboratively conducted by the Centers for Disease Control and Prevention (CDC). NHANES is designed to assess both the health and nutritional status of the civilian, noninstitutionalized population in the United States through a combination of comprehensive interviews, physical examinations, and laboratory tests. Ethical approval was secured from the CDC′s National Center for Health Statistics ethics board, with all study participants providing informed consent.

**Figure 1:**
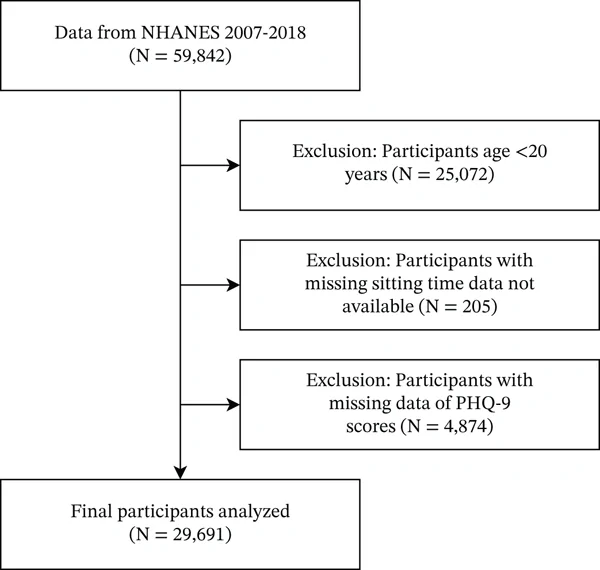
Participants selection flowchart.

Initially, the dataset included 59,842 participants. We excluded those under 20 years of age (*N* = 25,072) and individuals with missing information on sitting time (*N* = 205), along with participants lacking Patient Health Questionnaire‐9 (PHQ‐9) scores (*N* = 4874). This left a total of 29,691 participants in the final analytical sample. The selection process is shown in Figure 1.

### 2.2. Evaluation of Daily Sitting Time

The assessment of daily sitting time, a critical exposure variable in our study, was conducted using the well‐validated and widely used Global Physical Activity Questionnaire (GPAQ) [17]. Specifically, participants were asked, “On an average day, how many hours are you engaged in sitting or reclining activities?” This measurement encompasses all waking hours spent in a seated position, including time spent at work, home, or educational settings. Activities accounted for in this metric involve sitting at desks, socializing in static leisure contexts (like cafes or cinemas), commuting in vehicles such as cars, busses, or trains, as well as engaging in pastimes like reading, card playing, watching television, or using computers [18]. Responses that were refused or answered with “do not know” were classified as missing data, and the collected sitting time was recorded as minutes per day (min/d). Based on previous research [19–21], daily sitting time was converted from minutes to hours per day (h/d) by dividing by 60 for analysis. For analytical purposes, daily sitting time was classified into four distinct categories: fewer than 4 h, between 4 and 6 h, between 6 and 8 h, and 8 h or more. Notably, the reference group for comparison consists of participants reporting less than 4 h of sitting time daily.

### 2.3. Assessment of Depression

Depression was assessed using the PHQ‐9 [22]. The questionnaire consists of nine items that participants rate on a 4‐point scale ranging from 0 (*not at all*) to 3 (*nearly every day*), and the cumulative score can range from 0 to 27 [23]. A score of 10 or higher signifies the existence of depression, whereas a score under 10 implies no depression [24].

### 2.4. Assessment of Covariates

Covariates were identified in accordance with established research literature [25, 26], this study included extensive demographic, socioeconomic, and clinical variables. Including sex, marital status, race, education level, age, body mass index (BMI), alcohol consumption, poverty‐income ratio (PIR), smoking status, hypertension, moderate activity, and diabetes. Economic status is assessed via PIRs categorized as < 1.3, 1.3–3.5, and ≥ 3.5. BMI is classified into the following groups: ≤ 20, > 20 and ≤ 25, > 25 and ≤ 30, and > 30 kg/m^2^. Smoking status is evaluated based on whether participants have smoked at least 100 cigarettes in their lifetime. Alcohol consumption was computed by multiplying the self‐reported number of drinking days in the past year by the usual number of drinks consumed per drinking occasion, then dividing by 365 days. This continuous variable was subsequently categorized into ordinal levels: 0, > 0–≤ 1, > 1–≤ 2, and > 2 drinks/day [27, 28]. Hypertension is defined by a self‐reported diagnostic status, and moderate activity is recognized as engaging in at least 10 min per week of moderate‐intensity PA (e.g., brisk walking or cycling) [18]. Lastly, diabetes status is also based on self‐reports from participants. Detailed measurement protocols for these variables are publicly available at http://www.cdc.gov/nchs/nhanes/.

### 2.5. Statistical Analysis

In line with the NHANES analytical guidelines, the complex survey design was accounted for in the weighted analyses through the appropriate use of sampling weights, clustering, and stratification variables [29]. Baseline characteristics are summarized using survey‐weighted statistics, with continuous variables presented as means (95% CIs) and categorical variables as percentages (95% CIs). Referring to previous population‐based literature [30, 31], three models were employed to analyze the relationship between daily sitting time and depression. Model 1 was implemented without adjusting for covariates. Model 2 adjusted for confounding factors including age, sex, and race. Model 3 included more comprehensive adjustments for additional variables such as smoking status, marital status, hypertension, education level, PIR, alcohol consumption, BMI, moderate PA, and diabetes status. To examine the association between sitting time and depression, we conducted multivariate regression analyses. Potential nonlinear relationships were visualized using a generalized additive model (GAM) with smooth curve fitting [32, 33]. More specifically, to assess potential nonlinear associations, we fitted a two‐piecewise (segmented) logistic regression with the breakpoint selected by maximum likelihood and 95% CIs obtained via bootstrap [34, 35]. Additionally, sensitivity analyses were also undertaken to examine the strength of the results. We employed two strategies to handle missing covariate data: applying Multiple Imputation by Chained Equations (MICE) [36] and creating dummy variables for covariates with > 5% missing observations [37]. Subsequently, the primary model was re‐estimated using the newly generated imputed datasets in combination with sampling weights and design variables. Statistical analyses were carried out utilizing R software (Version 4.2) along with EmpowerStats. A significance threshold of *p* < 0.05 (two‐tailed) was established as the criterion for statistical significance in all analyses.

## 3. Results

### 3.1. Characteristics of Study Participants

This study included a total of 29,691 participants, of whom 9.2% (*n* = 2726) had a PHQ‐9 score of ≥ 10 (Table 1). Analysis of baseline characteristics revealed that, except for age (*p* = 0.1337), all other sociodemographic and health‐related factors showed significant differences between the depression and nondepression groups (*p* < 0.0001). The depression group had a higher proportion of females (64.52% vs. 50.11%), lower educational attainment (25.57% vs. 14.37% with less than high school education), lower income (41.55% vs. 19.69% with a PIR of < 1.3), and a higher rate of living alone (31.07% vs. 17.29% widowed/divorced/separated). Regarding health behaviors and conditions, the depression group reported longer daily sitting time (6.51 h vs. 6.18 h, *p* = 0.0067), higher prevalence of obesity (47.95% vs. 36.76% with BMI > 30), hypertension (43.96% vs. 31.41%), and diabetes (16.11% vs. 9.45%), as well as a lower proportion engaging in moderate PA (29.97% vs. 47.37%). Additionally, current smoking was significantly more common in the depression group (61.26% vs. 42.99%).

**Table 1:** Baseline characteristics of the study participants weighted.

Characteristics	Overall (*n* = 29,691)	No depression (PHQ‐9 < 10) (*n* = 26,965)	Depression (PHQ‐9 ≥ 10) (*n* = 2726)	*p*
Age, (years)	47.61 (47.14, 48.08)	47.67 (47.17, 48.17)	46.94 (46.09, 47.78)	0.1337
Sitting time/day (h)	6.21 (6.12, 6.30)	6.18 (6.09, 6.27)	6.51 (6.28, 6.74)	0.0067
Gender, (%)				< 0.0001
Male	48.73 (48.12, 49.35)	49.89 (49.24, 50.54)	35.48 (33.17, 37.85)	
Female	51.27 (50.65, 51.88)	50.11 (49.46, 50.76)	64.52 (62.15, 66.83)	
Race, (%)				< 0.0001
Mexican American	8.48 (7.11, 10.08)	8.52 (7.15, 10.12)	8.04 (6.30, 10.19)	
Other Hispanic	5.78 (4.90, 6.81)	5.58 (4.73, 6.57)	8.06 (6.44, 10.05)	
Non‐Hispanic White	67.07 (64.23, 69.79)	67.40 (64.56, 70.12)	63.27 (59.38, 67.00)	
Non‐Hispanic Black	11.03 (9.67, 12.55)	10.84 (9.49, 12.35)	13.25 (11.37, 15.38)	
Other race	7.64 (6.87, 8.50)	7.67 (6.86, 8.55)	7.39 (6.26, 8.70)	
Education level, (%)				< 0.0001
Less than high school	15.27 (14.14, 16.47)	14.37 (13.26, 15.56)	25.57 (23.20, 28.09)	
High school or GED	23.12 (22.04, 24.24)	22.84 (21.75, 23.97)	26.38 (24.21, 28.67)	
Above high school	61.61 (59.74, 63.44)	62.79 (60.91, 64.64)	48.05 (45.10, 51.01)	
Marital status, (%)				< 0.0001
Married/living with partner	63.24 (61.94, 64.53)	64.67 (63.36, 65.95)	46.97 (44.34, 49.61)	
Widowed/divorced/separated	18.40 (17.67, 19.16)	17.29 (16.57, 18.04)	31.07 (29.02, 33.20)	
Never married	18.35 (17.21, 19.56)	18.04 (16.83, 19.31)	21.96 (20.24, 23.78)	
PIR, (%)				< 0.0001
< 1.3	21.44 (20.10, 22.84)	19.69 (18.46, 20.98)	41.55 (38.36, 44.81)	
1.3–3.5	35.64 (34.34, 36.97)	35.59 (34.24, 36.97)	36.23 (33.61, 38.95)	
≥ 3.5	42.92 (40.88, 44.98)	44.72 (42.69, 46.77)	22.22 (19.34, 25.38)	
BMI, (%)				< 0.0001
≤ 20	4.67 (4.33, 5.03)	4.57 (4.21, 4.95)	5.81 (4.67, 7.21)	
> 20, ≤ 25	24.88 (23.97, 25.82)	25.26 (24.31, 26.24)	20.50 (18.43, 22.74)	
> 25, ≤ 30	32.79 (31.93, 33.66)	33.40 (32.49, 34.33)	25.74 (23.56, 28.03)	
> 30	37.66 (36.55, 38.79)	36.76 (35.59, 37.96)	47.95 (45.20, 50.72)	
Hypertension, (%)				< 0.0001
Yes	32.41 (31.33, 33.52)	31.41 (30.30, 32.54)	43.96 (41.32, 46.63)	
No	67.59 (66.48, 68.67)	68.59 (67.46, 69.70)	56.04 (53.37, 58.68)	
Diabetes, (%)				< 0.0001
Yes	9.99 (9.49, 10.51)	9.45 (8.96, 9.97)	16.11 (14.40, 17.98)	
No	87.79 (87.23, 88.33)	88.41 (87.85, 88.95)	80.69 (78.64, 82.59)	
Borderline	2.22 (2.00, 2.46)	2.13 (1.91, 2.38)	3.20 (2.27, 4.49)	
Moderate activity, (%)				< 0.0001
Yes	45.97 (44.52, 47.42)	47.37 (45.91, 48.83)	29.97 (27.16, 32.94)	
No	54.03 (52.58, 55.48)	52.63 (51.17, 54.09)	70.03 (67.06, 72.84)	
Smoking status, (%)				< 0.0001
Yes	44.46 (43.22, 45.70)	42.99 (41.76, 44.22)	61.26 (58.24, 64.20)	
No	55.54 (54.30, 56.78)	57.01 (55.78, 58.24)	38.74 (35.80, 41.76)	
Alcohol consumption, (%)				< 0.0001
0	3.92 (3.48, 4.42)	3.79 (3.33, 4.32)	5.51 (4.31, 7.01)	
> 0, ≤ 1	78.10 (77.10, 79.07)	78.31 (77.28, 79.30)	75.58 (72.79, 78.17)	
> 1, ≤ 2	10.75 (10.07, 11.48)	10.95 (10.22, 11.73)	8.34 (6.76, 10.25)	
> 2	7.22 (6.72, 7.77)	6.95 (6.42, 7.53)	10.57 (8.81, 12.62)	

*Note:* For continuous variables: survey‐weighted mean (95% CI), *p* value was by survey‐weighted linear regression. For categorical variables: survey‐weighted percentage (95% CI), *p* value was by survey‐weighted chi‐square test. Among the 29,691 participants, education level had 23 (0.08%) missing values, marital status had 17 (0.06%) missing values, poverty income ratio had 2661 (8.96%) missing values, BMI had 292 (0.98%) missing values, smoking status had 14 (0.05%) missing values, alcohol use had 8739 (29.43%) missing values, total moderate activity had five (0.02%) missing values, diabetes had 18 (0.06%) missing values, and hypertension had 37 (0.12%) missing values.

Abbreviations: BMI, body mass index; PHQ, Patient Health Questionnaire; PIR, poverty‐income ratio.

### 3.2. Association Between Daily Sitting Time and Depression

Table 2 illustrates the association between daily sitting time and the risk of depression. In the fully adjusted Model 3, each additional hour of sitting was associated with a 5% increase in the risk of depression (95% CI: 1.02, 1.07, *p* = 0.0002). When categorizing daily sitting time, participants who sat for 8 h or more daily exhibited a significantly higher risk of depression across all models. In Model 3, this group exhibited an odds ratio (OR) of 1.37 (95% CI: 1.12, 1.69, *p* = 0.0039) when compared with the reference group that sat for less than 4 h. In contrast, there were no statistically significant differences in depression risk between participants who sat for 4–6 h or 6–8 h and those in the reference group who sat for less than 4 h. The P for trend across sitting time categories was 0.0184 in Model 1, 0.0061 in Model 2, and 0.0025 in Model 3, supporting a significant positive dose‐response trend between sitting time and outcome risk.

**Table 2:** Univariate and multivariate analyses by logistic regression model weighted.

Exposure	Model 1	Model 2	Model 3
	OR (95% CI) *p*	OR (95% CI) *p*	OR (95% CI) *p*
Sitting time/day (h)	1.03 (1.01, 1.05) 0.0058	1.03 (1.01, 1.05) 0.0014	1.05 (1.02, 1.07) 0.0002
Group (h)			
< 4	1.0	1.0	1.0
≥ 4, < 6	0.93 (0.80, 1.08) 0.3457	0.95 (0.82, 1.11) 0.5184	1.04 (0.85, 1.27) 0.7352
≥ 6, < 8	0.97 (0.82, 1.15) 0.7287	1.01 (0.85, 1.19) 0.9335	1.03 (0.83, 1.28) 0.7733
≥ 8	1.16 (1.00, 1.35) 0.0501	1.20 (1.03, 1.40) 0.0190	1.37 (1.12, 1.69) 0.0039
P for trend	0.0184	0.0061	0.0025

*Note:* Model 1 was conducted without adjustments; Model 2 included adjustments for variables such as race, age, and sex; whereas Model 3 further incorporated adjustments for smoking status, marital status, hypertension, education level, PIR, alcohol consumption, BMI, moderate activity, and diabetes.

### 3.3. Analysis of Curve Fitting and Threshold Effects

**Figure 2:**
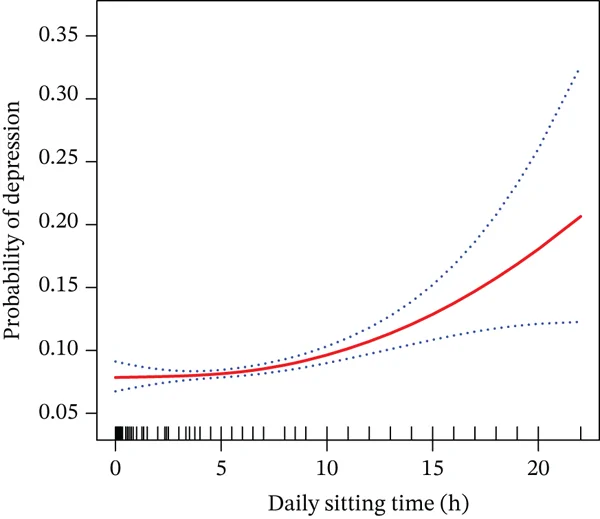
The red curve illustrates the smoothed fit of the variables, and the blue lines indicate the 95% confidence intervals enveloping this fit.

A threshold effect analysis employing a piecewise linear regression model clarified the nonlinear relationship between daily sitting time and the risk of depression (Table 3). The identified inflection point for daily sitting time was 7 h. Below this threshold, each additional hour of sitting was associated with a neutral risk of depression (OR 1.01, 95% CI 0.96, 1.07, *p* = 0.6272). In contrast, when daily sitting time exceeded the threshold, there was a significant increase in the risk of depression, with an OR of 1.07 (95% CI 1.01, 1.12, *p* = 0.0184). Results from the log‐likelihood ratio test showed that the two‐piecewise linear model outperformed the linear model significantly in describing the relationship between sitting time and the risk of depression (*p* = 0.010) (Figure 2; Table 3).

**Table 3:** Analysis of the threshold effect between daily sitting time and the risk of depression weighted.

Threshold effect analysis	Depression
	OR (95% CI) *p*
Inflection point of sitting time/day (K)	7
< K slope	1.01 (0.96, 1.07) 0.6272
> K slope	1.07 (1.01, 1.12) 0.0184
Log‐likelihood ratio test	0.010

*Note:* Adjustment factors included sex, age, race, education level, marital status, PIR, BMI, smoking status, alcohol consumption, hypertension, moderate activity, and diabetes.

To confirm the stability of our results, we performed a sensitivity analysis incorporating two strategies for handling missing covariate data: MICE and the use of dummy variables for covariates with > 5% missing observations. We continued to observe that our core findings remained stable, revealing a nonlinear relationship between daily sitting time and depression (Tables S1, S2, S3, S4, S5, and S6).

## 4. Discussion

Our investigation uncovered a significant nonlinear link between daily sitting duration and depression risk. Specifically, every extra hour spent sitting elevated depression risk by 5%, with a marked surge in individuals sitting for eight or more hours daily. Threshold effect analysis pinpointed 7 h as the critical juncture. Prior to this point, adding an hour of sitting did not significantly impact depression risk (OR 1.01, 95% CI 0.96, 1.07, *p* = 0.6272). However, once daily sitting time surpassed this threshold, the risk of depression rose considerably, with an OR of 1.07 (95% CI 1.01, 1.12, *p* = 0.0184).

A wealth of research has thoroughly examined the intricate links between sedentary behavior and depression. Central to this body of literature is the consistent finding that increased sedentary time correlates with heightened risks of mental health issues, emphasizing a dose–response relationship; that is, as sedentary time increases, so does the risk of developing depression [3, 38, 39]. Comprehensive reviews and meta‐analyses underscore this connection, noting not only the direct relationship but also the complex interplay between sedentary behavior and other lifestyle factors, such as PA, which can modulate the effects of prolonged sitting [40, 41]. Building upon this evidence, our study reveals identifying a nonlinear relationship between daily sitting time and depression risk, with an observed inflection point at approximately 7 h per day. Notably, our analysis indicates that sitting for durations below this point was not associated with a statistically significant increase in depression risk. This observation reinforces previous research indicating that moderate amounts of sedentary time may not necessarily lead to increased depressive symptoms [42, 43]. Conversely, beyond this duration, our results demonstrated a statistically significant elevation in depression risk. This aligns with conclusions drawn from multiple studies, illustrating that while moderate levels of sedentary behavior are associated with a higher risk of depression, excessive sedentary patterns may not show a proportional increase in risk, hinting at potentially nonlinear dynamics in this interaction [44, 45]. The divergence between our results and those of prior studies that have assumed a linear relationship may relate to various methodological differences, including study design, demographic variations within populations studied, and the ways in which sedentary behavior and depression are quantified [7, 46].

Several mechanisms may elucidate the nonlinear relationship between daily sitting time and depression risk. Although the present study′s data identify the nonlinear association itself, the following explanatory mechanisms are drawn from the established literature. A sedentary lifestyle contributes to decreased physical fitness and increases in BMI, both of which are established in the literature to correlate with heightened risk for depression [47, 48]. Research by Miao et al. indicates that the association between loneliness and negative health outcomes is stronger than that of social isolation, suggesting that loneliness exacerbates physical health issues, which can, in turn, influence mental health [49]. Additionally, evidence suggests that prolonged sitting can disrupt circadian rhythms and reduce exposure to natural light, which is critical for mood regulation [48]. Disturbances in circadian rhythms have been linked to mood disorders. Studies have shown that long periods of sedentary activity can result in mood dysregulation, thereby increasing vulnerability to depressive symptoms [48]. Furthermore, the social and psychological dimensions of prolonged sedentary behavior also require consideration. Extended periods of sitting are often linked to diminished social interaction and increased solitary activities, which can lead to social isolation and loneliness—well‐known contributors to depression [50–52]. The study by Christiansen et al. highlights that a lack of PA can significantly impact mental health, further establishing sedentary behavior as a pathway to psychological distress [53]. Additionally, isolative behaviors linked to excessive sitting patterns can exacerbate depressive symptoms, particularly among vulnerable populations [54, 55].

This study has several strengths. First, it offers new insights into the nonlinear relationship between daily sitting time and depression risk, providing a more nuanced understanding of this association. Second, the use of a comprehensive, nationally representative sample from NHANES contributes to the broader applicability of our results, allowing for more accurate estimates of the relationship between sitting time and depression in the US adult population. Even with these strengths, there are limitations associated with our study. First, the cross‐sectional design precludes the establishment of a temporal relationship and causal inferences between sitting time and depression. Longitudinal studies with repeated measures are needed to further explore this relationship. Second, PA is dichotomized as “≥ 10 min/week of moderate intensity,” which may not adequately control for confounding or mediation by PA. Furthermore, sitting time is a single self‐report item susceptible to recall and digit‐preference biases. Future research should consider objective measures (e.g., accelerometers) to improve accuracy. Additionally, defining depression as PHQ‐9 ≥ 10 is reasonable, but because the study is cross‐sectional and the outcome is not rare, ORs may overstate associations. Future prospective cohort studies employing objective measures of sedentary behavior are warranted to validate these findings and elucidate underlying mechanisms.

## 5. Conclusions

This study establishes a nonlinear association between daily sitting time and depression risk in US adults. This finding calls for future research to further explore the underlying mechanisms and causal relationships.

**Nomenclature**
NHANES: National Health and Nutrition Examination Surveys
PHQ: Patient Health questionnaire
WHO: World Health Organization
PA: physical activity
CDC: Centers for Disease Control and Prevention
GPAQ: Global Physical Activity Questionnaire
BMI: body mass index
PIR: poverty‐income ratio
GAM: generalized additive model
MICE: Multiple Imputation by Chained Equations

## Author Contributions

Z.C., H.C., and R.J. contributed equally to this work. Z.C., H.C., and R.J.: writing—original draft, visualization, software, methodology, investigation, formal analysis, data curation, and conceptualization. H.P. and Y.L.: writing—review & editing, validation, methodology, data curation, and conceptualization. J.L., Y.Y., and S.C.: writing—review & editing, methodology, and conceptualization. Y.W., R.L., and M.C.: writing—review & editing and conceptualization. W.C.: writing—review & editing, validation, supervision, resources, project administration, and funding acquisition.

## Funding

This study was supported by Shenzhen Second People′s Hospital Clinical Research Fund of Shenzhen High‐level Hospital Construction Project (Grant No. 2023yjlcyj027).

## Disclosure

All authors participated in discussions and revisions of the manuscript, ultimately reviewing and approving the final version submitted.

## Ethics Statement

All study participants provided informed consent, and the study protocol was approved by the Ethics Review Board of the National Center for Health Statistics (NCHS). Information can be found on the NHANES website (https://www.cdc.gov/nchs/nhanes/participant.htm).

## Consent

The authors have nothing to report.

## Conflicts of Interest

The authors declare no conflicts of interest.

## Supporting information


**Supporting Information** Additional supporting information can be found online in the Supporting Information section. Table S1: Threshold effect analysis between daily sitting time and the risk of depression: A sensitivity analysis after handling missing values using the indicator variable method. Tables S2–S6: Threshold effect analysis between daily sitting time and the risk of depression: A sensitivity analysis after handling missing values using multiple imputation.

## Data Availability

The publicly available datasets were analyzed in this study. These data can be found at: NHANES (https://wwwn.cdc.gov/nchs/nhanes/default.aspx).
