# Supplementary material for: Nonlinear Association of Daily Sitting Time With Depression Risk in US Adults: A Cross‐Sectional Study
Source: Behav Neurol. 2026 Mar 4;2026:7167267. doi: 10.1155/bn/7167267 (PMC13580794; doi:10.1155/bn/7167267)
Supplement: Supplementary file 1 [file BN-2026-7167267-s001.docx]

**Non-linear association of daily sitting time with depression risk in US adults: A cross-sectional study**

**Zhimao Cai^1,2,^*****, Hualang Cai^2,3,^*****, Rui Jia^1,^*****, Hui Peng^1^, Youlian Luo^1^, Jiashuang Lin^1,2^, Ye Ye^1^, Sixia Chen^1^, Yueqian Wang^4^, Rourou Li^1,2^, Mengjia Chen^1,2^, Weifeng Chen^1,✉^**

^1^Department of General Medicine, Shenzhen Second People’s Hospital, The First Affiliated Hospital of Shenzhen University, Shenzhen, 518000, Guangdong, China.

^2^Shenzhen University Health Science Center, Shenzhen, 518037, Guangdong, China.

^3^Department of Hepatology, National Clinical Research Center for Infectious Diseases, The Third People's Hospital of Shenzhen, Shenzhen, 518112, Guangdong, China.

^4^Yanchuan Communiy Health Service Center Songgang People’s Hospital Baoan District, Shenzhen, 518105, Guangdong, China.

*These authors contributed equally to this work.

**Correspondence:** Weifeng Chen**^1, ✉^**, chenweifeng2015@163.com

**Core Findings Indicator-variable Approaches**

Table S1 Analysis of the threshold effect between daily sitting time and the risk of depression-weighted.

| Threshold effect analysis | depression |
| --- | --- |
|  | OR (95%CI) p-value |
| Inflection point of Sitting time/day (K) | 7 |
| <K slope | 1.01 (0.97, 1.05) 0.6825 |
| >K slope | 1.07 (1.02, 1.12) 0.0095 |
| Log-likelihood ratio test | 0.011 |

Dummy variables were employed to indicate missing covariate values when the proportion of missing values exceeded 5%. Adjustment factors included sex, age, race, education level, marital status, PIR, BMI, smoking status, alcohol consumption, hypertension, moderate activity, and diabetes.

**Core Findings Following Multiple Imputation**

Table S2 Analysis of the threshold effect between daily sitting time and the risk of depression-weighted.

| Threshold effect analysis | depression |
| --- | --- |
|  | OR (95%CI) p-value |
| Inflection point of Sitting time/day (K) | 7 |
| <K slope | 1.01 (0.97, 1.05) 0.6424 |
| >K slope | 1.06 (1.02, 1.11) 0.0080 |
| Log-likelihood ratio test | 0.015 |

Adjustment factors included sex, age, race, education level, marital status, PIR, BMI, smoking status, alcohol consumption, hypertension, moderate activity, and diabetes. Sample size (n = 29691).

Table S3 Analysis of the threshold effect between daily sitting time and the risk of depression-weighted.

| Threshold effect analysis | depression |
| --- | --- |
|  | OR (95%CI) p-value |
| Inflection point of Sitting time/day (K) | 7 |
| <K slope | 1.01 (0.97, 1.05) 0.6198 |
| >K slope | 1.06 (1.02, 1.11) 0.0075 |
| Log-likelihood ratio test | 0.013 |

Adjustment factors included sex, age, race, education level, marital status, PIR, BMI, smoking status, alcohol consumption, hypertension, moderate activity, and diabetes. Sample size (n = 29691).

Table S4 Analysis of the threshold effect between daily sitting time and the risk of depression-weighted.

| Threshold effect analysis | depression |
| --- | --- |
|  | OR (95%CI) p-value |
| Inflection point of Sitting time/day (K) | 7 |
| <K slope | 1.01 (0.97, 1.05) 0.6556 |
| >K slope | 1.06 (1.02, 1.11) 0.0073 |
| Log-likelihood ratio test | 0.015 |

Adjustment factors included sex, age, race, education level, marital status, PIR, BMI, smoking status, alcohol consumption, hypertension, moderate activity, and diabetes. Sample size (n = 29691).

Table S5 Analysis of the threshold effect between daily sitting time and the risk of depression-weighted.

| Threshold effect analysis | depression |
| --- | --- |
|  | OR (95%CI) p-value |
| Inflection point of Sitting time/day (K) | 7 |
| <K slope | 1.01 (0.97, 1.05) 0.6164 |
| >K slope | 1.06 (1.02, 1.11) 0.0078 |
| Log-likelihood ratio test | 0.014 |

Adjustment factors included sex, age, race, education level, marital status, PIR, BMI, smoking status, alcohol consumption, hypertension, moderate activity, and diabetes. Sample size (n = 29691).

Table S6 Analysis of the threshold effect between daily sitting time and the risk of depression-weighted.

| Threshold effect analysis | depression |
| --- | --- |
|  | OR (95%CI) p-value |
| Inflection point of Sitting time/day (K) | 7 |
| <K slope | 1.01 (0.97, 1.05) 0.6500 |
| >K slope | 1.06 (1.02, 1.11) 0.0082 |
| Log-likelihood ratio test | 0.017 |

Adjustment factors included sex, age, race, education level, marital status, PIR, BMI, smoking status, alcohol consumption, hypertension, moderate activity, and diabetes. Sample size (n = 29691).
